# Exercise, Cardiovascular Health, and Risk Factors for Atherosclerosis: A Narrative Review on These Complex Relationships and Caveats of Literature

**DOI:** 10.3389/fphys.2020.00840

**Published:** 2020-07-31

**Authors:** Lucas P. Santos, Daniel Umpierre

**Affiliations:** ^1^Exercise Pathophysiology Laboratory, Clinical Research Center, Hospital de Clínicas de Porto Alegre, Porto Alegre, Brazil; ^2^National Institute of Science and Technology for Health Technology Assessment (IATS/HCPA), Hospital de Clínicas de Porto Alegre, Porto Alegre, Brazil; ^3^Graduate Program in Health Sciences, Cardiology and Cardiovascular Sciences, Faculty of Medicine, Universidade Federal do Rio Grande do Sul, Porto Alegre, Brazil

**Keywords:** exercise, physical activity, cardiovascular health, atherosclerosis, cardiovascular disease

## Abstract

The following narrative review addresses the relationship between physical activity and exercise with cardiovascular health, focusing primarily on the following risk factors for atherosclerosis: hypertension, dyslipidemia, and vascular function. Cardiovascular diseases are intimately associated with mortality and morbidity, and current societal organization contributes to the incidence of cardiovascular events. A worldwide epidemiological transition to cardiovascular deaths was observed in the last century, with important decrements in physical activity and diet quality. An atherogenic environment started to be the new normal, with risk factors such as dyslipidemia, hypertension, and endothelial dysfunction observed in great portions of the population. Exercise is an important tool to improve overall health. For hypertension, a great amount of evidence now puts exercise as an effective therapeutic tool in the treatment of this condition. The effects of exercise in modifying blood lipid–lipoprotein are less clear. Despite the rationale remaining solid, methodological difficulties impair the interpretation of possible effects in these variables. Vascular function, as assessed by flow-mediated dilatation, is a good measure of overall vascular health and is consistently improved by exercise in many populations. However, in individuals with hypertension, the exercise literature still needs a further description of possible effects on vascular function variables. Physical activity and exercise are associated with improved cardiovascular health, especially with reduced blood pressure, and should be encouraged on the individual and population level. Evidence regarding its effects on blood lipids and flow-mediated dilatation still need solid landmark studies to guide clinical practice.

## Introduction

Cardiovascular diseases (CVDs) are not only the main cause of death worldwide, accounting for around 20% of deaths, but also additionally impose relevant morbidity and disability to those non-fatally affected by them (Benjamin et al., 2019). Additionally, CVDs also cause an important financial burden, loss of quality of life for the families of those who survived an event, and are one of the most expensive conditions for public health spending due to its marked prevalence (Benjamin et al., 2019).

Contemporary societies face a pandemic of hard cardiovascular outcomes, and although the mortality of such events dropped (Ribeiro et al., 2016; Mensah et al., 2017), risk factors such as lack of physical activity (PA), hypertension, and dyslipidemia still remain important. In the plethora of preventive strategies for health, exercise stands out as one intervention that can potentially address multiple classical cardiovascular risk factors (Eckel et al., 2014; Arnett et al., 2019), whereas possibly also affecting various surrogate measures of cardiovascular risk, such as cardiac structure (Kokkinos et al., 1995), baroreflex sensitivity (Laterza et al., 2007), and vascular health (Ashor et al., 2015).

In the following review, we intend to explore the context of CVDs with an emphasis in specific risk factors, such as hypertension, dyslipidemia, and endothelial dysfunction, their relationship with PA and exercise, how the current state-of-art can support these interventions, and what are the next steps to enhance the scientific knowledge in this field. We chose to explore these factors due to their importance for CVDs, together with the interesting presentation of the literature.

## Epidemiologic Transition Causes and Consequences in Population Health

Modern societies have been going through many changes driven by technological advances. Some of these changes were so deep that these are now reflected not only in the way people live but also in how they die. A transition from deaths related to communicable diseases to those caused by non-communicable diseases was first observed in developed countries, amidst the last century, and is often termed as an “epidemiologic transition” (Omran, 2005; McKeown, 2009).

In low-to-middle-income countries (LMICs), between 1960 and 1980, CVDs became the leading cause of mortality (Frenk et al., 1989; Ribeiro et al., 2016). Yet, three of the five leading causes of years of life lost in 1990 were still related to poor sanitary and healthcare conditions (Frenk et al., 1989; Marinho et al., 2018). Data from Brazil in 2016 now show a shift toward non-communicable diseases. Moreover, conditions related to lifestyles such as ischemic heart disease, stroke, and diabetes have sharply gained importance, and CVDs are now responsible for more than 30% of mortality in this country (Ribeiro et al., 2016).

Urbanization and industrialization processes resulted in changes in PA and dietary intake. In developed countries, this was observed when work-related PA dropped (Church et al., 2011), whereas the consumption of high-fat and high-sugar foods had substantial growth (von Deneen and Liu, 2011). Nowadays, LMICs are no different, with rates of overweight and obesity, physical inactivity, and all health conditions attributable to these factors comparable with those in more developed nations.

## Physical Activity and Exercise as the Lost Link Between Lifestyle and Health

One key aspect of humans’ successful evolution was the ability to run long distances. As a societal hunter–scavenger, sustaining a high-paced pursuit for food was a strong evolutionary advantage (Lieberman et al., 2009), and therefore, our bodies were built to be physically active. As modernization advanced, these characteristics became less needed, and now, individuals spend less and less energy on a regular day.

Morris et al. were the first to show an association between PA and CVD when compared inactive and active workers, with a lower incidence of hard events shown in the latter. Since then, a great amount of evidence shows the benefits of PA. On the other hand, a decrease in occupational-related PA is noted, as demonstrated in a five-decade analysis (1960–2010), when a sharp increase in physically light [2.0–2.9 metabolic equivalents (METs)] and sedentary (<2.0 METs) work activities was coupled with plummeting levels of moderate activities (>3.0 METs) (Church et al., 2011).

Although prospective randomized studies evaluating the effects of PA on all-cause or cardiovascular mortality are non-existent, causative links between these factors are undeniable. One of the latest evidence to support such a claim is a recent meta-analysis by Ekelund et al. (2016) who found that high amounts of moderate-to-vigorous PA were associated with the elimination of the risk of death associated with high sitting time. This new evidence added importance to PA, showing that it could mitigate the associations of sedentary behavior with hard outcomes that were once thought independent. Conversely, evidence from an observational analysis of 354,277 employees between 18 and 75 years evaluated in occupational health screenings in Sweden showed a population decrease of 6.7% in absolute and 10.8% in relative cardiorespiratory capacity assessed by maximal oxygen consumption (Ekblom-Bak et al., 2019), showing that conditioning levels are dropping on a population level. Additional subgroup analyses showed that younger ages and men were the most affected strata.

Taken together, these facts reveal a “pandemic of physical inactivity.” A recent report, including 1.9 million participants, showed a 27.5% prevalence of insufficient levels of PA worldwide (Guthold et al., 2018). These numbers directly impact public health, with an attributable fraction to physical inactivity of 6% of coronary heart disease burden and 9% of premature deaths (Lee et al., 2012). This way, PA is now strongly recommended as a public health measure to diminish the impact of non-communicable diseases by many scientific societies and governmental institutions (World Health Organization, 2010; Eckel et al., 2014; Physical Activity Guidelines Advisory Committee, 2018).

## Atherosclerosis – The Disease of the Century

While hard cerebro/cardiovascular outcomes such as ischemic stroke or myocardial infarction can produce devastating consequences on their own, they have one silent and long-lasting underlying factor in common: a diseased artery. In these cases, susceptible sites on the vasculature have been going through a process of subendothelial lipoprotein retention, vascular wall inflammation, and plaque formation. This process can take decades until it peaks either with vessel stenosis (and manifestations such as angina) or with plaque rupture and subsequent cellular death. The etiologic process of cerebro/CVDs relies heavily on the complex pathophysiological process of atherogenesis (Tabas et al., 2007; Gimbrone and García-Cardeña, 2016).

In a post-mortem analysis of 2,876 subjects between 15 and 34 years old, Strong et al. (1999) showed that atherosclerotic lesions were present in all aortas within the youngest age strata (15–29 years) and that the extent and prevalence of such findings increased in the oldest age group (30–34 years). These results are corroborated by other autopsy-based (McGill et al., 2000) and imaging (Tuzcu et al., 2001) studies, demonstrating that the process of subendothelial fatty-streak accumulation begins early in life and tends to happen in every human. However, lipid trapping and accumulation are not sufficient to explain why this process tends to spin out of control, leading to plaque growth and possible destabilization. Ultimately, the establishment of atherosclerotic disease is multifactorial, consisting of different components (physical, inflammatory, immunologic, metabolic, and biochemical), all of which play a role in its development.

**FIGURE 1:**
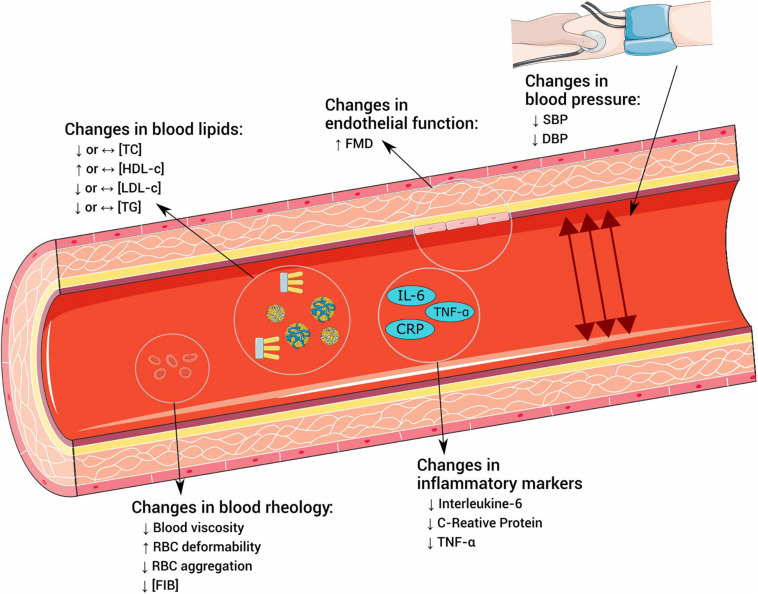
Effects of exercise in risk factors for atherosclerosis. TC, total cholesterol; HDL-c, high-density lipoprotein cholesterol; LDL-c, low-density lipoprotein cholesterol; TG, triglycerides; FMD, flow-mediated dilatation; SBP, systolic blood pressure; DBP, diastolic blood pressure; RBC, red blood cell; FIB, fibrinogen; IL-6, interleukin 6; CRP, C-reactive protein; TNF-α, tumoral necrosis factor α.

In the following sections, we will specifically address three of these factors: dyslipidemia, hypertension, and endothelial dysfunction, later exploring their interface with PA and exercise. We also briefly mention two other factors that pertain to the pathophysiology of atherosclerosis: blood rheology and inflammation. These factors were chosen due to their close relationship with atherogenesis and the potential to be affected by exercise. Figure 1 summarizes the effects of exercise on the explored outcomes.

## Dyslipidemia and the Roots of Atherosclerosis Etiology

An imbalance in lipoprotein serum levels can disrupt homeostasis and lead to pathological conditions in the cardiovascular system. The term dyslipidemia was coined for any metabolic state that denotes this imbalance and is used to classify several conditions affecting lipoprotein metabolism and that implies an increased risk of disease. Subendothelial infiltration of apoB-containing lipoproteins, such as low-density lipoprotein cholesterol (LDL-c), is the basis of atherogenic processes (Ference et al., 2017). If plasma levels of these molecules are elevated, there is an increased chance of their infiltration and retention in the vascular wall, initiating plaque formation. A robust review of mendelian randomized studies has shown a logarithmic risk reduction for coronary heart disease for individuals exposed to lower LDL-c levels through life, independently by which mechanism these lower LDL-c levels are achieved (Ference, 2015).

High-density lipoprotein cholesterol (HDL-c), however, as opposed to LDL-c and other atherogenic molecules, plays a protective role in atherosclerosis pathophysiology. The main antiatherogenic property of HDL-c seems to be related to macrophage cholesterol efflux in a process that leads to the removal of cholesterol from macrophages for subsequent transport to the liver (Rothblat and Phillips, 2010). Evidence shows that each increase in 1 mg/dl of HDL-c is related to 2–3% of CVD risk reduction (Gordon et al., 1989).

Triglyceride levels, despite having a less clear association with CVDs, also compose the commonly assessed “lipid profile” and are believed to be important in the pathophysiology of atherosclerosis. In fact, hypertriglyceridemia affects LDL-c and HDL-c composition and metabolism, resulting in a dysfunctional and more atherogenic lipid profile (Miller et al., 2011). Therefore, disturbances in the balance between lipid levels, as supported by the information mentioned earlier, result in augmented CVD risk, especially in lipid profiles presenting what is called the atherogenic dyslipidemic triad (high triglycerides and LDL-c and low HDL-c) (Nordestgaard et al., 2020).

### Interaction Between Lipids and Exercise: Clear as Mud

The physiological rationale for exercise interventions to positively alter the lipid–lipoprotein profile is clear. Several mechanisms could influence these changes, for example, improvements in the inflammatory profile, enhanced overall oxidative capability, and increased baseline and total daily energy expenditure. The logic behind this claim is such that exercise is indeed recommended as a tool for modifying serum lipids and to help address dyslipidemia (Riebe et al., 2018). Nonetheless, when it comes to the evidence regarding the effects of exercise training in modifying lipid–lipoprotein profile, there is still much debate. Several meta-analyses were published, with disagreeing results. We bring a non-exhaustive list of these studies to illustrate these disparate findings inside this literature.

Halbert et al. (1999), were pioneers in the synthesis of the effects of either aerobic or resistance exercise training on blood lipids profile. In their meta-analysis, 1,833 sedentary adults with no established disease were included in 31 trials (Halbert et al., 1999). The authors reported that aerobic exercise training was effective in modifying all measures of serum lipids evaluated [total cholesterol (TC): -3.9 mg/dl, LDL-c: -3.9 mg/dl, HDL-c: +1.9 mg/dl, and triglycerides: -7.1 mg/d]. However, these results should be interpreted with caution due to the limited clinical significance of the effect sizes reported and the high heterogeneity presented among the selected studies. In the same study, only four trials examining the effects of resistance training were included, with no differences in serum lipid associated with the exposure to this modality of exercise.

Hespanhol et al. (2015) examined the effects of running-based training on health markers of previously sedentary subjects. Their meta-analysis included 2,024 subjects, distributed in 35 studies. Regarding lipid–lipoproteins, the authors found a significant intervention effect only on HDL-c (+2.2 mg/dl) and triglycerides (-13.7 mg/dl), with no effects observed on TC or LDL-c. Murtagh et al. (2015) on the other hand, examined the effects of walking interventions on cardiovascular health outcomes, including lipid–lipoproteins, through a meta-analytical approach. Lipid–lipoprotein markers did not change with interventions. The results of a systematic review conducted by Tambalis et al. (2009) agree with the notion implied by the opposite effects in HDL-c in running and walking interventions observed earlier. In their review, the authors state that only 6 out of 28 trials evaluating moderate-intensity aerobic training showed a significant improvement in HDL-c, whereas 22 out of 37 trials of high-intensity aerobic training improved HDL-c. Therefore, a dose–response relationship on aerobic training intensity seems to exist in HDL-c responses to exercise, as higher intensities tend to elicit more favorable changes in this variable. However, this dose–response relationship is yet to be demonstrated experimentally.

The effects of resistance exercise training on lipid–lipoproteins were explored by Kelley & Kelley, on a reevaluation of a previous meta-analysis (Kelley and Kelley, 2009a), using an improved statistical approach (Kelley and Kelley, 2009b). The authors reported that despite observed improvements for TC, LDL-c and triglycerides, the prediction intervals calculated for a true effect in a new study in all variables pointed to a neutral effect, and therefore, caution is advised when recommending resistance exercise training to modify blood lipid profile.

Kelley & Kelley, in another meta-analytic synthesis, explored the effects of aerobic exercise training alone, diet alone, or the combination of these two approaches on serum lipid–lipoproteins. In the six included trials with direct comparisons between the interventions, exercise was not effective in modifying TC, LDL-c, and HDL-c but had a significant effect of -6.0 mg/dl in triglycerides. Diet alone was effective in reducing TC (-10.0 mg/dl), LDL-c (-5.3 mg/dl), and TG (-10.6 mg/dl), with no effect in HDL-c. On the other hand, combining diet with exercise was also not effective in modifying HDL-c values, whereas TC (-13.7 mg/dl), LDL-c (-8.8 mg/dl), and triglycerides (-13.3 mg/dl) were positively affected by this intervention (Kelley et al., 2012).

Taken together, these results demonstrate that the effectiveness of exercise training in lipid–lipoprotein balance remains debatable. Although the biological plausibility to this claim is still solid and evidence regarding impacts of aerobic exercise training intensity on HDL-c points to a possible dose–response effect, the literature on blood lipid–lipoprotein responses to exercise warrants further development. Therefore, as recommended by the current lipid management guidelines (Grundy et al., 2019), comprehensive behavior change, also encompassing dietary changes, might be the most reasonable lifestyle approach to address dyslipidemia.

## Hypertension – The Silent Companion of Cardiovascular Disease

Arterial blood pressure (BP) is the force exerted by the blood in any given unit area of the artery walls. It is the result, in terms of fluid mechanics, of the interaction between the heart pumping blood during each cardiac cycle and the resistance exerted by the arteries to the produced blood flow. Higher and sustained BP values characterize hypertension, and its prevalence exceeds 1.3 billion people worldwide – around 30% of the world’s adult population (Mills et al., 2016). Its prevalence increases with age, with pooled estimates pointing to figures around 60% among individuals older than 60 years (Mills et al., 2016). Hypertension is an important public health issue, as there is a strong association between BP levels and CVD, with data showing that each increase in 20 mm Hg in systolic BP (SBP) or 10 mm Hg in diastolic BP (DBP) doubles the risk for acute myocardial infarction or ischemic stroke (Lewington et al., 2002).

The multifactorial etiology of hypertension adds to the complexity of this health condition. Because the bodily processes involved in BP regulation derive from varied physiological systems and their interactions (i.e., nervous, humoral, cardiovascular, renal systems), maladaptation in any of these regulatory mechanisms can result in sustained elevated BP. Furthermore, chronic periods of high BP can also negatively impact these regulation processes, worsening BP levels even more. This vicious cycle sums up to the natural vascular aging process, resulting in a scenario of sympathetic hyperactivation (Grassi et al., 2015), impaired baroreflex sensitivity (Bristow et al., 1969), shear-stress-related endothelial insults (Davies, 2009), endothelial dysfunction (Mordi et al., 2016), and structural changes in the vascular system (Zanchetti et al., 1998) and the heart (Im et al., 1995). Its prevalence, imposed risk, and tendency of worsening with age and/or lack of control make hypertension the most relevant preventable cardiovascular risk factor in the contemporary ages (GBD, 2015; Risk Factors Collaborators, 2016). Yet, hypertension remains poorly treated in LMICs, with estimates of around only 10% of individuals with hypertension considered within controlled ranges of BP (Geldsetzer et al., 2019).

Recently, a robust randomized clinical trial designed to test intensive SBP treatment to a target below 130 mm Hg – against the usual target of 140 mm Hg – in hard cardiovascular outcomes was conducted. The trial had a premature ending because mortality rates were significantly different between groups, favoring intensive control (SPRINT Research Group et al., 2015). This new evidence prompted changes in hypertension guidelines in the United States, which now considers the cutoff points of 120 mm Hg for SBP and 80 mm Hg for DBP (Whelton et al., 2018). These changes result in 46% of the adult American population with at least hypertension stage I. The new European guidelines, however, did not follow the changes, and the cutoff for hypertension in Europe remains 140/90 mm Hg (Williams et al., 2018). Although the debate on cutoff points may persist in the following years, both guidelines agree when underscoring the importance of lifestyle changes, such as a healthy diet and exercise, as part of hypertension treatment. Those are accessible and effective measures that can help lower population BP levels, increase the quality of life, and lower public health expenses, especially in countries with limited resources and poor healthcare coverage.

### Exercise as Therapy to Hypertension

The acute, subacute, and chronic responses to exercise – as defined by immediate (at exercise onset), short-term (minutes to several hours after an exercise session), and long-term adaptations (weeks to months after exercise training commencement), respectively – are widely studied physiological effects in BP levels. These responses were described in many aspects of cardiovascular responses to effort, ranging from how immediate changes take place in the cardiovascular system when exercising, to potential morphological adaptations induced by chronic reductions in BP related to exercise. To date, exercise is well regarded as a key lifestyle tool for hypertension, mentioned and endorsed with the maximal grade of evidence in most guidelines for hypertension management (Malachias, 2016; Whelton et al., 2018; Williams et al., 2018).

#### Acute Effects of Exercise in Blood Pressure

Exercise onset implies in augmented cardiovascular demand. In aerobic and resistance exercises, there are increased needs for blood flow in the exercised muscle tissues, which raises heart rate and cardiac output – determinants of SBP. Therefore, raises in this variable are expected to a certain extent. On the other hand, DBP is more influenced by peripheral vascular resistance, which behaves differently among these two modalities, leading to maintenance or decreases in this variable with aerobic exercise and increases with resistance exercise for healthy individuals.

In individuals with hypertension, however, this hemodynamic behavior might not be so clear. Exaggerated BP responses to exercise are a common finding in this population and known to be related to cardiovascular maladaptations (Ren et al., 1985) and poorer prognostic (Mundal et al., 1994). Mechanisms inherent to hypertension pathophysiology such as sympathetic hyperactivity (Manolis et al., 2014) and increased peripheral vascular resistance (Mayet and Hughes, 2003) might influence these responses. Additionally, these observations are irrespective of treatment status, suggesting limited pharmacological effectiveness of antihypertensive medication regarding BP responses to physical efforts (Chant et al., 2018). Although no consensus on a clear definition has been established, values equal or above 200 mm Hg for SBP and/or 110 mm Hg for DBP during submaximal aerobic efforts can be considered exaggerated responses (Kokkinos, 2015).

#### Subacute Effects of Exercise in Blood Pressure

With descriptions dating more than 120 years (Hill, 1898), subacute exercise-induced hypotension [named post-exercise hypotension (PEH)] is vastly explored, mainly because it is thought to be the driving force behind chronic BP changes with exercise (Thompson et al., 2001). These subacute effects are produced in varied exercise settings and different populations. For example, Pescatello et al. (1991) demonstrated acute BP reductions that lasted for 12.7 h in a control-matched sample of men with hypertension exposed to an aerobic exercise session. Later, the same group demonstrated an apparent intensity dose–response effect in a mixed sample of 45 men with prehypertension and hypertension, where the more pronounced effects of BP reductions were found in the day when the exercise intensity was higher, as compared with moderate and low intensities (Eicher et al., 2010). Keese et al. (2011) showed the presence of PEH after different exercise modalities (aerobic, resistance, and combined), with shorter durations observed in resistance exercise sessions when compared with aerobic or combined. Rondon et al. (2002) in an experiment with acute exercise and ambulatory BP monitoring, demonstrated that PEH is also observed in older adults with hypertension and can persist for 22 h. Moreover, we have described that individuals with resistant hypertension, despite their pharmacological unresponsiveness, also exhibit PEH for 19 h in ambulatory BP monitoring evaluations after an aerobic exercise session. In this population, however, lower intensities seemed to be more efficient in acutely reducing BP values when compared with moderate intensities (Santos et al., 2016).

These subacute BP responses to exercise are derived from changes in hemodynamic regulation, and its mechanisms are not fully elucidated to date. In fact, there is evidence showing the effects of exercise in many different aspects of BP regulation (Halliwill et al., 2013), which can ultimately lead to BP reductions. Some of the known mechanisms behind these acute responses involve a compensatory sympathetic withdrawal (Kulics et al., 1999), baroreflex resetting (Halliwill et al., 1996), and peripheral vascular resistance reduction (Cléroux et al., 1992) – as a possible consequence of sustained histamine-induced vasodilation (Barrett-O’Keefe et al., 2013) – coupled with possible changes in cardiac output after an exercise session (Rondon et al., 2002). More importantly, these reductions are closely related to chronic decreases in BP related to exercise training. A prospective interventional study evaluating 17 middle-aged individuals with prehypertension explored the relationship between exercise-induced acute and chronic BP changes, showing that the magnitude of subacute reductions may predict the extent of chronic BP lowering after training (Liu et al., 2012). This close relationship raises the hypothesis that chronic exercise-induced BP reductions are an expression of a summation of recent acute exercise effects in BP values (Thompson et al., 2001).

#### Chronic Effects of Exercise in Blood Pressure

Chronic exposure to exercise directly impacts BP values. The quantity and quality of evidence available to make this claim are such that many meta-analytic estimates show the positive chronic effects of various exercise modalities in BP (Cornelissen and Smart, 2013; Corso et al., 2016; MacDonald et al., 2016; Wu et al., 2019).

Cornelissen and Smart (2013) conducted one of the most robust meta-analytic explorations of chronic exercise effects on BP values. The authors explored the effects of exercise training in different modalities on BP parameters of individuals with varied categories of BP (normotension, prehypertension, and hypertension). The authors included in their analysis 93 randomized clinical trials that lasted ≥4 weeks, totaling 5,223 patients in 153 intervention groups. Exercise type was divided into endurance, resistance, combined, and isometric resistance training (IRT). Their most compelling finding was that for individuals with hypertension, aerobic exercise training could imply a reduction of 8.2 mm Hg for SBP and 5.2 mm Hg for DBP. On the other hand, no significant BP reduction was observed in the other modalities, probably due to the inclusion of fewer studies in those arms in comparison with aerobic training.

Recently, however, the current paradigm that exercise prescription for hypertension needed to be focused on aerobic exercise and only complemented by other types of exercise started to be challenged. MacDonald et al. conducted a meta-analysis evaluating 64 controlled studies (*n* = 2,344) to determine the efficacy of resistance training as a sole therapy to modify BP values (MacDonald et al., 2016). With the same approach, Corso et al. investigated 68 controlled studies (*n* = 4,110) examining the effects of concurrent training (i.e., combining resistance and aerobic training) on the same outcome (Corso et al., 2016). The authors of both meta-analyses found that, in individuals with higher baseline BP values, either resistance or combined resistance and aerobic training can be effective in chronically reducing BP values with an effect between 5 and 6 mm Hg for both SBP and DBP. With current evidence, it is safe to say that chronic exercise training in either aerobic or resistance modalities can be effective tools in modifying BP values and can be used as therapeutic tools for hypertension. These new pieces of evidence prompted a change in the current recommendations of exercise prescription for hypertension from the American College of Sports Medicine, which now considers both modalities to target BP (American College of Sports Medicine, 2019; Pescatello et al., 2019).

Other modalities of exercise might also impact BP. Wu et al. (2019) recently conducted a meta-analysis on the effects of yoga training on BP. In their pooled analysis of 49 trials, yoga was more effective than control in reducing SBP and DBP values of individuals with prehypertension (-5.2 mm Hg for SBP and -2.8 mm Hg for DBP) and hypertension (-8.7 mm Hg for SBP and -4.8 mm Hg for DBP). These results were even more pronounced in those yoga interventions with breathing and meditation components. Yet, the authors warn that the methodological quality of the included studies is low, and because of this fact, the confidence assigned to the meta-analysis results is suboptimal.

More recently, motivated by the previously observed potential effect of IRT (Cornelissen and Smart, 2013), a new meta-analysis using the “individual patient data” approach (Smart et al., 2019) examined the effects of this intervention in BP values. Using a robust methodology, the authors evaluated 12 trials, with 14 intervention groups, totaling 326 patients (191 enrolled in IRT and 135 in control), analyzed at the individual level. The authors showed reduction effects from 6.2 to 7.3 mm Hg for SBP and 2.8 to 3.3 mm Hg for DBP favoring IRT. While interesting and promising, several points should also be taken into consideration while interpreting these results. It is important to notice that this meta-analysis had mixed samples of individuals with and without hypertension, with 52% of the total samples receiving antihypertensive medication. IRT-induced adaptations might overlap with the physiological effects of antihypertensive medication (Millar et al., 2014), bearing a potential hindering-effect in the benefit for individuals treated for hypertension. Also, the total sample size of 326 patients, coupled with the information discussed earlier, demonstrates that more robust trials, evaluating individuals with hypertension solely and exploring the interactions with medications, are still warranted to improve the evidence of IRT as a treatment for hypertension.

Adding to the earlier discussed evidence on the importance of exercise in the management of hypertension, Naci et al. (2018) in a robust network meta-analysis indirectly comparing more than 39.000 subjects, demonstrated that exercise effects are comparable with those produced by common antihypertensive drugs in SBP. In their pioneer analysis, the authors have shown that for individuals with SBP >140 mm Hg, both pharmacological and exercise interventions present a similar reduction effect of approximately 9 mm Hg. In this context, it is clear that exercise training is a notably efficient tool as a non-pharmacological therapy for hypertension. Despite the known limitations of network approaches in meta-analytic studies, the presented findings are novel and exciting. At the same time, they might bear the potential to increase the importance given to exercise as an antihypertensive therapy (Pescatello, 2019).

## Endothelial Dysfunction Close Relationship With Hypertension and Cardiovascular Disease

A significant aspect of cardiovascular health is closely related to endothelial function. The vascular endothelium, located in the intimal portion of the vascular wall, is responsible for secreting a myriad of vasoactive molecules, playing a key role in vasomotor balance. Likewise, these cells are also involved in a series of physiological processes such as the regulation of coagulation/anticoagulation cascades, inflammatory/anti-inflammatory activity, immunologic responses, and morphological remodeling (Konukoglu and Uzun, 2017). Because of this important role in vascular homeostasis, impairments in endothelial cell function are a critical aspect in the pathophysiology of atherogenesis (Gimbrone and García-Cardeña, 2016), and therefore, the assessment of endothelial function was used to describe several populations of interest.

Endothelial vasodilatory function, mediated mostly by nitric oxide (NO) release, is considered one of the endothelium’s most relevant physiological modulations and can be assessed directly or indirectly in various forms. With the advance biomedical sciences, developments of techniques, such as the catheter-based angiography, venous occlusion plethysmography, and ultrasound imaging of flow-mediated dilatation (FMD), allowed the assessment of the endothelium-mediated vascular motricity. Together with these techniques, physiological studies allowed the role of endothelial cells to be more well elucidated with the understanding of NO metabolism and its correlates (L-arginine, nitrites, and nitrates) (Kelm, 1999) and the important role of NO modulation by NO synthase on vascular regeneration (Heiss et al., 2010).

The use of high-definition ultrasound is now preferred in vascular function evaluation due to its reduced costs and easiness to perform, when compared with catheter-based assessments, and its accuracy when compared with venous occlusion plethysmography. Nowadays, other techniques, such as finger plethysmography and peripheral artery tonometry, have also been described (Nohria et al., 2006; Matsuzawa et al., 2010).

The endothelial function assessment performed by ultrasonographic imaging of FMD, commonly performed after an occlusion maneuver in the brachial artery, is considered a proxy of general vascular health. Evidence from a comprehensive meta-analysis of prospective observational studies showed that increased FMD is correlated with reduced risk for cardiovascular outcomes in both non-CVD and CVD populations. This pooled estimate of more than 17,000 patients, followed between 6 and 115 months, demonstrated that each increase of 1% in FMD is related to a 12% risk reduction for cardiovascular outcomes (Matsuzawa et al., 2015). Interventions that positively alter endothelial function might bear the potential of protecting against future cardiovascular events, although controlled clinical studies prospectively evaluating changes in FMD and its associations with cardiovascular outcomes are lacking in the literature.

Endothelial dysfunction is widely associated with hypertension. Panza et al. (1990), on an early observation of this relationship, compared the responses to acetylcholine of forearm blood flow and vascular resistance of patients with hypertension with those of normal controls, showing impaired endothelial responsiveness in hypertension. Similarly, Treasure et al. (1993) showed impaired endothelium-dependent coronary vasodilation in subjects with hypertension when compared with normotensive controls. Additionally, vascular repair seems to be also impaired in hypertension, as shown by a cross-sectional evaluation of 160 subjects, demonstrating that aging and hypertension are associated with a lower number of circulating endothelial progenitor cells (Umemura et al., 2008). Although the understanding if endothelial dysfunction is a consequence or a cause of hypertension is not clear, both conditions indicate poor cardiovascular health, and strategies to address either one might bear the potential to affect the other.

### Exercise Impacts on Endothelial Function

Even before the Nobel-winning discovery of the endothelial vasodilatory function (Furchgott and Zawadzki, 1980) and the later understanding of the role of NO and shear-stress on this endothelial-derived vasodilation, the potential of exercise to modify vascular function sparked great interest. Early experiments using indirect measures of vascular function (i.e., venous occlusion plethysmography) were pioneers to demonstrate peripheral hemodynamic behavior during and after exercise (Barendsen, 1973).

Nowadays, the notion that exercise can improve vascular function is well-established. Mechanisms of these changes are related to short-term positive adaptations in NO bioavailability and regulation by endothelial NO synthase that can ultimately lead to vascular remodeling and sheer normalization (Green et al., 2004). These mechanisms counteract the vascular maladaptation related to aging and should be considered as a first-line approach to vascular dysfunctions (Tanaka, 2019). In an example of these claims, a pooled analysis of 51 randomized controlled trials on the effects of exercise training in different modalities (aerobic, resistance, or combined) on FMD showed that all examined types of exercise could be effective in improving vascular function (Ashor et al., 2015). In this analysis, the mean effect sizes observed were among 2–3% increases in FMD for all modalities.

On the other hand, sedentary behavior is associated with impaired endothelial function. Quasi-experimental data show that 5 days of bed rest (Hamburg et al., 2007; Nosova et al., 2014) or, in the data from a crossover trial, even prolonged sitting for periods as low as 3 h (Thosar et al., 2015) can immediately affect vascular function in healthy individuals. Additionally, in the referred trial, 5-min walks as breaks in sedentary behavior prevented the decrease of vascular function associated with prolonged sitting (Thosar et al., 2015). Taken together, these results show how quickly physical inactivity can impair vascular function and how PA and exercise can contribute to mitigate these effects.

In patients with a history of CVD, such as coronary artery disease and heart failure, exercise interventions are demonstrated to restore endothelial function. Hambrecht et al. evaluated the coronary artery function of patients with coronary artery disease exposed to a 4-week high-frequency (daily) exercise training program compared with a control group receiving usual care (Hambrecht et al., 2000). Arterial function was assessed through drug-infusion angiographies. The patients in the exercise group improved coronary vascular function as expressed by a 54% smaller acetylcholine-induced vasoconstriction, whereas no changes were observed in the control group. The same author also demonstrated similar benefits in patients with heart failure exposed to an exercise intervention. When compared with non-exercising controls, these patients showed enhanced vascular function as expressed by a 203% increase in peripheral blood flow in response to acetylcholine (Hambrecht et al., 1998).

In individuals with hypertension, however, it is not clear whether exercise can be effective in improving vascular function. A recent meta-analysis, including five trials in individuals with hypertension exposed to aerobic exercise, found a +1.5% (95% confidence interval of -0.11 to +3.0%) improvement in FMD values (Pedralli et al., 2018). These results are indicative of a possible increase that still needs confirmation in future studies due to the neutral effects pointed by the confidence intervals.

Westhoff et al. (2007) evaluated a 12-week, 3 days/week program of walking-based interval training in variables of cardiovascular health, including FMD, in older adults with isolated systolic hypertension, compared with a sedentary control group. The authors reported a difference in the variation of pre–post FMD among the study arms, with the exercise group expressing an increase in FMD values of 2.3%. Interestingly, the pre–post difference in FMD values did not achieve statistical significance (*p* = 0.43). It is unknown, however, if these findings are generalizable to those with regular hypertension, who might have different impairments in their vascular control.

The current state-of-art challenges the notion that endothelial function impairments are easily reversible in samples with a dysfunctional vasculature. In individuals with hypertension, for example, the degree of vascular maladaptation can be such that exercise interventions in common research settings (i.e., short-term, small sample sizes) might not be enough to elicit verifiable improvements in these parameters. As endothelial dysfunction is associated with cardiovascular risk factors and chronic conditions, more studies with robust sample sizes and designs are needed to better understand the effects of exercise on vascular function of populations with different health conditions.

## Blood Rheology, Inflammation, and the Role of Exercise

Despite this review’s focus on the risk factors for atherosclerosis mentioned earlier, two other factors deserve a brief mention: blood rheology and inflammation. Both are crucial to atherogenesis and are potentially affected by exercise interventions. More comprehensive reviews on both topics can be found in the work of others (Brun et al., 1998; Toth et al., 2007; Libby, 2012; Connes et al., 2013).

Blood viscosity, determined by hematocrit, plasma viscosity, and red blood cell (RBC) profile, is associated with cardiovascular risk factors (Koenig and Ernst, 1992), incident events (Sweetnam et al., 1996), and disease severity (Kesmarky et al., 1998). Biophysical and flow-related changes in the blood are observed in arterial bifurcations and bends. By disrupting endothelial function and structure, these events facilitate lipid trapping and platelet adherence, especially in scenarios of increased blood viscosity and fibrinogen levels. Additionally, resultant decreases in flow velocity allow conformational changes in RBC, promoting further RBC aggregation (Toth et al., 2007). Evidence shows that hemorheological markers of atherogenesis can be positively affected by increased PA and exercise in healthy populations, in those at risk for CVDs and in secondary prevention (Romain et al., 2011; Sandor et al., 2014). These practices are associated with lower blood viscosity and hematocrit, as well as RBC deformability (Brun et al., 1998). Additionally, reductions in plasma viscosity and RBC aggregation, mainly through decreased fibrinogen levels, are also observed in individuals exposed to higher PA and in patients with CVD exposed to exercise interventions (Brun et al., 1998; de Meirelles et al., 2014). However, high-quality randomized clinical trials exploring these parameters in exercise interventions are still scarce.

Inflammation is key to all steps of atherogenesis. The endothelial impairments mentioned earlier trigger the adhesion and infiltration of monocytes and T cells. The uptake of lipid molecules by macrophages in the subendothelial space then leads to the formation of foam cells. From there, an intense cross talk, mediated by immune, endothelial, and smooth muscle cells, and pro-atherogenic cytokines [mainly interleukins (IL) 1 and 6 and tumoral necrosis factor α] (Tedgui and Mallat, 2006), beyond the scope of this review, ensures a positive feedback loop for further plaque development and destabilization (Libby, 2012). Epidemiological studies demonstrate clear associations between levels of inflammatory markers, especially C-reactive protein (CRP), and the incidence of CVD (Emerging Risk Factors Collaboration et al., 2012), strengthening the so-called “inflammatory hypothesis” for atherogenesis. Cumulative evidence from robust placebo-controlled clinical trials targeting lipid-lowering therapies (Nissen et al., 2004; Ridker et al., 2008) and, more recently, the Canakinumab Anti-inflammatory Thrombosis Outcome Study trial (Ridker et al., 2017), targeting IL-1β for reducing cardiovascular events, now serve as a proof-of-concept on the possible impact of improving inflammatory markers on future cardiovascular events and mortality. Exercise interventions have been shown as potential mediators of improved inflammatory profiles. A recent meta-analysis of randomized controlled trials, evaluating 1,138 healthy middle-aged and older patients, showed improvements in tumoral necrosis factor α, IL-6, and CRP levels in individuals exposed to aerobic exercise training, when compared with those in non-exercising controls (Zheng et al., 2019). These results are following a previous meta-analysis evaluating the effects of exercise on CRP levels that, in sensitivity analyses, also showed reduced concentrations of this inflammatory marker in patients with established CVD and type 2 diabetes (Fedewa et al., 2017).

## Final Remarks

PA and exercise are undeniably tied to improved cardiovascular health in varied scenarios. Interventions aiming to increase the time people spend in these activities can have positive impacts on individual and population health. Nonetheless, the exercise literature still needs further development to improve the understanding of whether exercise can be used to enhance specific markers of health. This fact is ultimately related to the methodological characteristics of the literature. A common caveat observed is that some areas (i.e., effects of exercise on lipids and FMD) still lack a body of literature comprised of robust landmark studies, with enough quantity and quality to draw more definite conclusions on the potential of such interventions. On the other hand, however, exercise is better described as an effective treatment for hypertension. Although improvements in this area of knowledge are still needed, this fact alone should be sufficient for stakeholders to stimulate the implementation of such practices in the public health context, especially in LMICs.

## Author Contributions

LS developed the conceptual framework and drafted the manuscript. DU suggested topics, discussions, references, and items, as well as revised the manuscript. Both authors contributed to the article and approved the submitted version.

## Conflict of Interest

The authors declare that the research was conducted in the absence of any commercial or financial relationships that could be construed as a potential conflict of interest.
